# Social support, psychological flexibility and coping mediate the association between COVID-19 related stress exposure and psychological distress

**DOI:** 10.1038/s41598-022-12262-w

**Published:** 2022-05-23

**Authors:** Richard Tindle, Alla Hemi, Ahmed A. Moustafa

**Affiliations:** 1 School of Health and Behavioural Sciences, Discipline of Psychology, University of the Sunshine Coast, Sunshine Coast, QLD Australia; 2 School of Education, Bar Ilan University, Ramat Gan, Israel; 3 Faculty of Society and Design, Bond University, Gold Coast, QLD Australia; 4 The Faculty of Health Sciences, Department of Human Anatomy and Physiology, School of Psychology, University of Johannesburg, Johannesburg, South Africa

**Keywords:** Psychology, Human behaviour

## Abstract

The COVID-19 pandemic has contributed to an increase in psychological distress. However, protective factors such as social support, psychological flexibility, and coping mechanisms can help individuals cope with the effects of psychological distress. This study aimed to test a recent hypothesis suggesting that psychological flexibility is not necessarily a coping strategy but a mechanism that can influence the coping strategies an individual employs during stressful events. We tested a mediation model that COVID-19 concerns would contribute to higher levels of perceived social support, which would directly increase psychological flexibility, and finally test if the effect of psychological flexibility on distress was mediated by approach and avoidant coping strategies. The results show that social support facilitates higher levels of psychological flexibility. Further, that psychological flexibility indirectly reduces psychological distress by reducing avoidant coping and increasing approach coping strategies. Within the context of COVID-19, we have shown the importance of social support and psychological flexibility for reducing distress. We have provided further evidence that psychological flexibility might not be a coping mechanism but a strategy that leads individuals to engage in more approach coping strategies and fewer avoidant coping strategies.

## Introduction

The novel Coronavirus Disease 2019 (COVID-19) pandemic has caused worldwide disruption and contributed to an increase in psychological distress among the general public^1^. An initial study found that 35% of Chinese respondents reported high levels of psychological distress and nearly 5% showed severe levels of psychological distress. Davillas and Jones^2^ found that the prevalence of psychological distress in a UK sample increased from 18.5 to 27.7% during the first wave of the COVID-19 pandemic. In Spain, psychological distress was prevalent in 65.2% of respondents^3^. In Australia, approximately 33% of participants reported high to very high levels of psychological distress^4^, and in Italy, 43.4% of respondents presented with moderate levels of psychological distress and 5.3% reported severe levels of psychological distress. There is evidence emerging from multiple countries, ethnicities, and demographics that there has been a significant increase in the psychological distress of individuals due to COVID-19. Therefore, it is important to identify which psychological and social factors can help protect against psychological distress caused by COVID-19.

### Social support

Social support from friends, family, colleagues, and others is important for well-being, reducing psychological distress, and improving quality of life^5–9^. Conversely, when individuals do not have access to social support, levels of stress are often higher and individuals experience more symptoms associated with depression and anxiety^10^. Establishing and seeking social support during stressful events is an important protective factor that helps reduce the effects of negative psychological outcomes^11^.

In individualistic cultures (e.g., Australia, Canada, UK, and the USA), people are more likely to explicitly seek social support from friends, family, and colleagues during stressful events, to receive assistance, consolation, and assurance^6,12–15^. Explicit social support means actively seeking advice, assistance, and emotional support from others^16^; and is associated with a reduction in negative psychological consequences linked to stressful events^6,13,14,16^. Although social support is important for enhancing psychological wellbeing, it is not necessarily the size of a person’s social support network but the quality and frequency of the social support they receive that is protective against psychological distress^17^.

During the COVID-19 pandemic, governments have enforced regulations such as strict social distancing, quarantining, or self-isolation. As a result, there have been reports of a reduction in social support and increased feelings of loneliness among the general population^1,9^. Social isolation is also linked to poor mental health and higher levels of psychological distress^10,18^. Intuitively, we might argue that the circumstances related to COVID-19’s social isolation and quarantining contribute to a reduction in social support and a subsequent increase in feelings of loneliness and distress.

Bu and colleagues^19^examined predictors of loneliness during the pandemic in a large sample (*n* = 35,000) and discovered that perceived social support was an important protective factor against loneliness. However, the authors found that the government policies and regulations enforcing social distancing were not associated with feelings of loneliness. Instead, the strongest predictor of loneliness was an individual’s baseline measure of loneliness. This suggests that individuals who did not have established, frequent, and quality social support before COVID-19 are at a greater risk during lockdown periods. These results highlight two important points. First, frequent and quality social support networks are important for coping with the stressors associated with COVID-19. Second, social support does not necessarily need to be face-to-face. For example, those who had pre-established social support networks before COVID-19 successfully attained social support during the pandemic, even in conditions of social distancing. That is, social support can be maintained through online services and social media with the same effectiveness as face-to-face contact ^20–22^.

### Psychological flexibility

Psychological flexibility is the ability to maintain focus on the current situation, and employ values-based behaviour even during difficult and stressful events^23,24^. Francis et al.^25^ described three core interconnected components of psychological flexibility, within the framework of acceptance and commitment therapy: openness to experience, behavioural awareness, and valued action^25,26^. Openness to experience refers to the willingness to accept pleasant and unpleasant feelings associated with the current circumstances. Behavioural awareness refers to being aware of thoughts, behaviours, and actions, and identifying their purpose. Valued action refers to engaging in thoughts and behaviours that align with what the individual deems meaningful and important. In the context of acceptance and commitment therapy, being open to accepting feelings, being aware of those feelings, and persisting to change behaviour based on values and goals despite negative feelings, are said to improve psychological flexibility^25,26^.

High levels of psychological flexibility are associated with better coping, acceptance, adjustment, and improved wellbeing^23,27,28^. During stressful life events, psychological flexibility can protect individuals from negative feelings, allow the person to adjust to negative situations, improve mental health^29^, and reduce the impact of undesirable psychological outcomes^30,31^.

Kroska and colleagues^24^investigated how psychological flexibility contributes to reducing psychological distress during COVID-19. Results of hierarchical regressions showed that high levels of psychological flexibility were protective against psychological distress. However, despite high levels of psychological flexibility amongst their sample, the impact of COVID-19 related concerns remained a significant predictor of psychological distress. These results indicate that while psychological flexibility can help reduce psychological distress, it is not enough on its own to completely remove the negative psychological impacts caused by COVID-19.

Another relevant point is the possible mediating role of psychological flexibility between COVID-19 related stress and psychological problems^32^. In addition to a direct relationship between psychological inflexibility and the development of depression, anxiety, and distress^24,32–34^, psychological flexibility also mediates and moderates the negative psychological impacts of COVID-19 related stressors^34–36^. Specifically, psychological flexibility is protective when an individual can accept their feelings, accept the negative consequences of COVID-19, and persist in changing their behaviour based on values and goals despite the current circumstances^25,26^. Conversely, when individuals are rigid and unwilling to adapt to social changes, they are likely to be more psychologically inflexible and at risk for high levels of psychological distress and poor mental health and wellbeing. When an individual is psychologically flexible, the negative impacts of COVID-19 on psychological distress can be mitigated or reduced.

### Coping

Coping refers to our ability to deal with stressors^37^. In the context of this research, we refer to two broad coping mechanisms that are typically implemented by individuals—avoidant coping and approach coping. Avoidant coping refers to coping behaviours that are maladaptive and include self-distraction, denial, substance use, behavioural disengagement, venting, and self-blaming^38^. Avoidant coping styles usually result in an increase in psychological distress and a reduction in psychological well-being and quality of life^38^. Approach coping is characterised by adaptive behaviours such as emotional support, instrumental support, positive reframing, planning, and acceptance^38^.

### Avoidant coping

Avoidant coping strategies often include actions that allow an individual to ignore, get away from, or use distraction to avoid unpleasant thoughts caused by a stressor in their environment. Avoidant coping is a maladaptive coping strategy that is associated with lower levels of psychological flexibility^39^ and has been linked to higher levels of depression, anxiety, and distress^40–42^. Leonidou and colleagues^39^ investigated the moderating role of psychological flexibility and avoidant coping on the quality of life of individuals with psychosomatic symptoms. Their results confirmed that there is a negative relationship between psychological flexibility and avoidant coping. Further, avoidant coping strategies were associated with higher levels of anxiety and lower quality of life. Their study concluded that identifying avenues to reduce the use of avoidant coping strategies could alleviate anxiety-related symptoms.

Conversely, some studies have found that avoidant coping strategies can also be useful to cope with stressors^43^. For example, emotion-focused coping strategies might include relaxation, forms of escapism, substance use, or ignoring the stressor^44–46^. These strategies are typically good for short-term relief and can sometimes minimize the emotional reactivity to a stressor and provide an individual relief to think about how to effectively deal with a stressor^46^. However, avoidant coping strategies are typically not useful for long-term coping as they do not effectively target the cause of the stress and can lead to maladaptive coping strategies (e.g., substance abuse). Indeed, during the COVID-19 pandemic, avoidant coping strategies have been typically associated with higher levels of depression, anxiety, and distress^47–54^ and there does not appear to be any evidence to show that employing an avoidant coping strategy during the COVID-19 pandemic would produce positive or useful outcomes for an individual’s psychological wellbeing.

### Approach coping

Approach coping strategies include behaviours that allow an individual to focus on finding a solution to environmental stressors. These can include cognitive focussed strategies that involve active coping (i.e., controlling or removing the stressor), acceptance of the stressor, positive reframing, and psychological growth. There is some evidence within the literature that positive coping is associated with higher psychological flexibility, and lower levels of negative psychological outcomes (e.g., depression, anxiety, and distress)^55,56^. Indeed, during the COVID-19 pandemic, approach coping strategies have been associated with lower levels of psychological distress^54,55,57^. However, as pointed out in a recent study^58^, there is a lack of research into the association between psychological flexibility (and inflexibility) and coping styles.

### Coping and psychological flexibility

Rueda and Valls^58^ investigated the mediating role of coping strategies between psychological *inflexibility* and the quality of life of patients with psychological disorders. Coping styles measured in their study included all 14 subscales of the Brief COPE^59^; including those related to avoidant coping (i.e., self-distraction, denial, substance use, behavioural disengagement, venting, and self-blaming), approach coping (i.e., active coping, use of emotional and instrumental support, positive reframing, planning, and acceptance), humour, and religion. The results showed that participants who engaged in avoidant coping strategies such as denial, venting, behavioural disengagement, and self-blame were also more psychologically inflexible. Self-distraction was the only avoidant coping strategy not associated with psychological inflexibility. In terms of approach coping strategies, only positive reframing and acceptance were associated with lower psychological inflexibility. Further, their mediation analyses found that psychological inflexibility indirectly increased anxiety and depression through denial coping strategies (i.e., avoidant coping), while acceptance (i.e., approach coping) mediated the relationship between psychological inflexibility and depression. These results indicate that psychological inflexibility indirectly impacts psychological well-being depending on the type of coping strategy utilised by an individual. Their results showed that when an individual is psychologically inflexible, they are more likely to use avoidant coping strategies which exacerbate their anxiety, depression, and distress.

Dawson and Golijani-Moghaddam^33^ investigated how avoidant and approach coping styles mediated the relationship between psychological inflexibility and psychological wellbeing, anxiety, depression, and distress during the COVID-19 pandemic. Their results identified that psychological inflexibility indirectly increased psychological distress and decreased psychological wellbeing when an individual employed an avoidant coping style. In their analysis, they also found a direct relationship between psychological inflexibility and distress. These results suggest that psychological inflexibility can result in more avoidant coping styles which further exacerbates psychological distress. Dawson and Golijani-Moghaddam^33^ argue that psychological flexibility might not be a coping mechanism but might determine the type of coping mechanism an individual will employ during stressful events. For example, individuals with high levels of psychological flexibility will employ more approach coping strategies and reduce avoidant coping strategies. On the other hand, individuals with low levels of psychological flexibility are likely to utilise more avoidant coping strategies and fewer approach coping strategies. In the context of COVID-19, understanding the association between psychological flexibility and coping mechanisms can better identify ways to protect against COVID-19 related distress.

### Social support and psychological flexibility

Despite social restrictions, evidence has shown that individuals have continued to maintain and seek social support from friends and family by utilising online services and social media, during the COVID-19 pandemic. We argue that social support can increase our perception of how well we can cope with the current pandemic, increase psychological flexibility, and improve mental health. The idea that social support is directly associated with psychological flexibility has been understudied—with only one other study using an Omanian sample identifying a positive association between psychological flexibility and social support^60^.

Psychological flexibility seems to mediate the relationship between COVID-19 stressors and our overall levels of psychological distress. There are important clinical and therapeutic implications for acknowledging the impact of psychological flexibility during a global pandemic. As the COVID-19 situation is out of the control of the individual, social support and psychological flexibility could be important protective factors that can lead to an increase in adaptive coping mechanisms (i.e., approach coping) and a reduction in maladaptive coping mechanisms (i.e., avoidant coping). As social support and psychological flexibility are known to protect against psychological distress caused by negative events; it is plausible that receiving social support from friends and family will contribute to an increase in psychological flexibility, an increase in approach coping mechanisms, a reduction in avoidant coping mechanisms, and a reduction in psychological distress.

### Study aims

**Figure 1:**
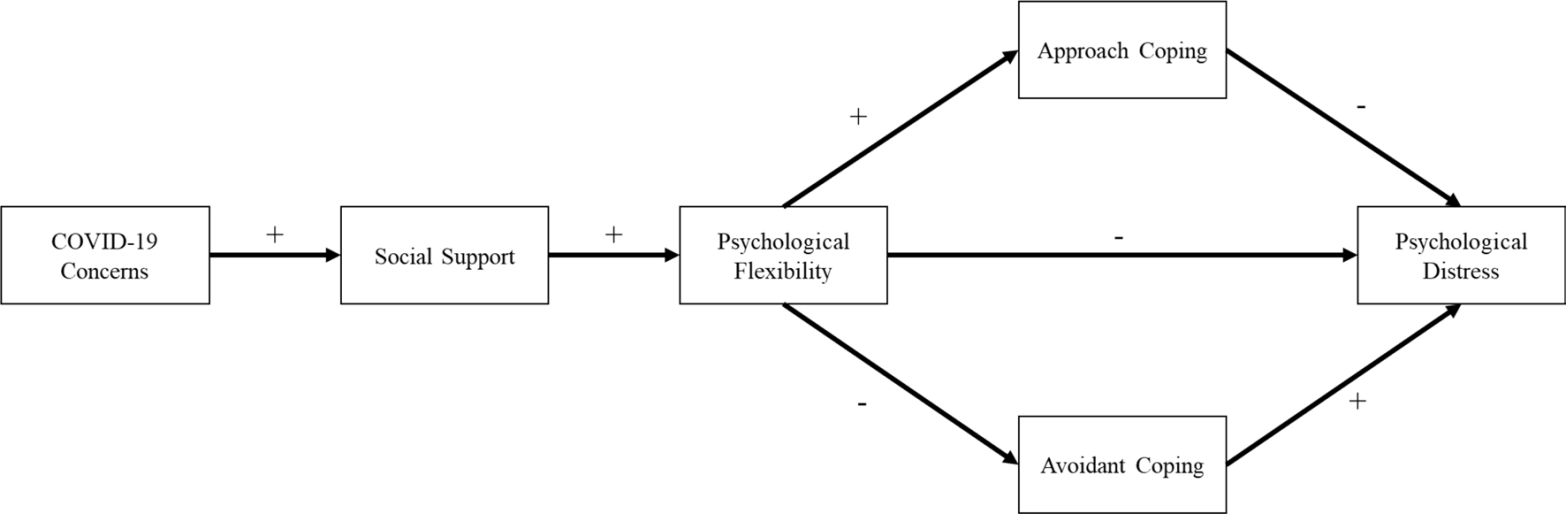
The hypothesised model to identify the pathway from COVID-19 concerns and psychological distress through social support, psychological flexibility, approach coping, and avoidant coping. Positive and negative signs indicated the predicted association between each variable.

This study aims to test a model (Fig. 1) of the pathway between experiencing stressful events (i.e., COVID-19) and psychological distress through social support, psychological flexibility, and coping mechanisms. It is hypothesised that there will be a direct positive association between COVID-19 concerns and social support. That is, during stressful events, individuals are likely to seek more social support. There will be a direct positive association between perceived social support and increased levels of psychological flexibility. For psychological flexibility, we hypothesise that high levels of psychological flexibility will be associated with an increase in approach coping strategies and a reduction in avoidant coping strategies. Finally, we hypothesise that psychological flexibility and approach coping will be negatively associated with psychological distress (i.e., a reduction in psychological distress) and avoidant coping will be positively associated with psychological distress. In terms of indirect effects, we hypothesise that COVID-19 concerns will indirectly impact psychological flexibility through social support, psychological flexibility, and coping styles. Specifically, we hypothesise that psychological flexibility will indirectly reduce psychological distress through avoidant coping and approach coping. That is, psychological flexibility will reduce psychological distress by first reducing avoidant coping strategies and increasing approach coping strategies.

## Method

### Participants

A Monte-Carlo simulated power analysis for a serial mediation model (^61^,20,000 simulations, 1 – β = 0.90, r = 0.50) determined a minimum sample size of 345 was needed to detect the indirect and direct effects of our mediation analysis. In total, 360 participants began the survey, five participants did not complete any questions, and four participants had missing data greater than 10% and were removed from the analysis – the data was deemed missing at random. Excluding the data had no bearing on the results. The total sample size used in the data analysis was enough to detect the indirect effects (*n* = 351). The age of participants ranged from 18 – 88 (*M* = 37.06, *SD* = 15.29), participants identified as female (*n* = 265, 74.40%), male (*n* = 55, 15.40%), or identified as a gender minority (e.g., transgender, non-binary, a-sexual; *n* = 28, 7.80%) and a majority of the sample identified as Caucasian (i.e., White/European; *n* = 294, 82.6%).

### Materials

The survey was administered using Qualtrics and participants completed demographic questions, the Kessler 10 Psychological distress Scale (K10)^62^, The Comprehensive Assessment of Acceptance and Commitment Therapy Processes (CompACT)^25^, the Brief COPE^38,59^; Multidimensional Scale of Perceived Social Support^63^; and the COVID -19 Concerns Scale. All scales were presented using a counterbalanced sequence for each participant.

#### COVID-19 concerns scale

The Trauma, Coping, & Growth Lab at Bar-Ilan University, Israel has recently developed a scale to measure concerns during the COVID-19 pandemic. Participants are asked to identify their level of agreement with each statement within the context of the COVID-19 pandemic. For example, participants are asked how strongly they agree with the following statement “*I feel stressed more than usual since the outbreak of COVID-19*” or “*The COVID-19 pandemic is a good opportunity for strengthening the value of solidarity* “. The COVID-19 concerns scale showed excellent reliability (McDonald’s ω = 0.83, 95%, CI [0.80, 0.85]).

#### The multidimensional scale of perceived social support

The multidimensional scale of perceived social support (MSPSS) is a reliable and valid measure that has been used more than 8000 times in studies measuring social support^63^. The measure comprises 12 items and is scored on a 7-point Likert scale (1-strongly disagree – 7 strongly agree). The maximum score on the scale is 84 with higher scores indicating higher perceived social support. This questionnaire measures social support from family (e.g., *My family really tries to help me*), friends (e.g*., I can talk about my problems with my friends*), and a significant other (e.g., *There is a special person who is around when I am in need*). The MSPSS showed excellent generalisable reliability (McDonald’s ω = 0.91, 95%, CI [0.89, 0.92]).

#### The comprehensive assessment of acceptance and commitment therapy

The Comprehensive Assessment of Acceptance and Commitment Therapy processes (CompACT)^25^ is a reliable and valid measure that comprises three components of psychological flexibility: openness to experience, behavioural awareness, and valued action^25^. The CompACT comprises 23 items rated on a 7-point Likert scale (0 “strongly disagree” to 6 “strongly agree”). The question included statements related to resilience such as, “*I try to stay busy to keep thoughts or feelings from coming*” and “*I undertake things that are meaningful to me, even when I find it hard to do so”*. Higher scores indicate greater openness to experience, behavioural awareness, and valued action. The CompACT showed excellent generalisable reliability (McDonald’s ω = 0.91, 95% CI [0.90, 0.93]).

#### The Brief COPE

The Brief COPE^59^ is a valid and reliable 28-item measure of coping styles. Each item is rated using a 4-point Likert scale (1 “I haven’t been doing this at all” to 4 “I’ve been doing this a lot”). Eisenberg et al.^38^ conducted a factor analysis of the Brief COPE and identified two broad coping strategies: (1) avoidant coping (comprising self-distraction, denial, substance use, behavioural disengagement, venting, and self-blaming); and (2) approach coping (comprising active coping, use of emotional and instrumental support, positive reframing, planning, and acceptance). To calculate the total for avoidant coping and approach coping, the items relating to religion and humour are excluded because they comprise both adaptive and maladaptive strategies^38,64^. In recent literature, the Brief COPE has been used to identify avoidant and approach coping strategies within similar samples and during the COVID-19 pandemic^53,55,65–67^. Further, the broad categories of approach coping, and avoidant coping have been replicated in multiple samples (see^4,33,64^). For example, questions include items focussed on approach coping (e.g., *“I've been trying to come up with a strategy about what to do*”) and avoidant coping (e.g., “*I've been getting emotional support from others”, I've been saying to myself "this isn't real."*). In the current study, the Brief COPE was calculated using Eisenberg and colleagues’ (2012) scoring to capture approach and avoidant coping styles. Within our sample, the avoidant coping sub-scale showed good reliability (McDonald’s ω = 0.81, 95% CI [0.77, 0.83]) and the approach coping sub-scale showed excellent reliability (McDonald’s ω = 0.87, 95% CI [0.85, 0.89]).

#### The Kessler psychological distress scale

The Kessler Psychological Distress Scale (K10;^68^ is a ten-item measure of how often the respondent has felt symptoms of depression and anxiety in the previous four weeks. Participants respond to each item by indicating if they have experienced a symptom none of the time, a little of the time, some of the time, most of the time, or all the time, such as “*about how often did you feel tired out for no good reason?*” and “*about how often did you feel depressed?*”. The K10 is validated, and normative data are available for Australia^69^. The K10 scores are categorised as low (10–15), moderate (16–21), high (22–29), and very high (30–50) psychological distress^70^. The K10 showed excellent generalisable reliability (McDonald’s ω = 0.94, 95% CI [0.94, 0.95]).

### Procedure

The study was approved by the Charles Sturt University Human Research Ethics committee (H20297). All methods were carried out in accordance with relevant guidelines and regulations specified by the Human Research ethics committee. Participants were recruited through Charles Sturt University, Western Sydney University, and from the public. Charles Sturt University and Western Sydney University were recruited by asking colleagues to post announcements on subject sites and through first-year participation pools. Students were also recruited by posting on student social media groups (e.g., Facebook, Reddit, Instagram, and Twitter), word of mouth, and snowballing. Participants from the public were recruited through social media advertising (i.e., Facebook and Instagram), snowballing techniques, and word of mouth. Recruitment remained open until the desired sample size was reached.

Participants were invited to complete a 15 minute survey. Participants were informed that the questions would ask them for demographic information (e.g., gender, age, relationship status, cultural background) as well as questions about their social support, psychological distress, coping strategies, and their ability to overcome stressful situations (e.g., COVID-19). After reading the information sheet, participants were informed that by clicking the “next button” to begin the survey they were consenting (i.e., informed consent) to participate in the research. This form of consent was approved by the Charles Sturt University Human Research Ethics Committee and is consistent with acceptable forms of consent outlined in Sect. 2.2.5 of the Australian Governments National Statement on Ethical Conduct in Human Research “*Consent may be expressed orally, in writing or by some other means (for example, return of a survey, or conduct implying consent)*”. After beginning the survey, the order of scales (e.g., COVID-19 concerns, Brief Cope, MSPSS, and CompACT) was randomised for each participant. However, all participants completed demographic questions at the end of the survey. Once participants completed the survey, they received a debrief statement and were thanked for their participation.

### Statistical analysis

A serial-parallel mediation model using 20,000 Monte-Carlo simulated bootstrapping was conducted using AMOS to test the hypothesised model (Fig. 1). Model fit was assessed using the adjusted goodness of fit index (AGFI), root means squared error of approximation (RMSEA), and the Tucker Lewis Index (TLI or NFI). For the AGFI, a value ≥ 0.90 is recognised as an acceptable level of good fit^71,72^. For RMSEA, lower non-significant values are considered a better fit. In terms of cut-off values, RMSEA values, ≤ 0.07 are considered to indicate a good fit^72,73^. Recommendations for TLI indicate that values ≥ 0.95 should be used to indicate a good model fit^72^. Non‐significant χ^2^ p‐values were another measure of model fit^74^. However, it should be noted that when using robust maximum likelihood and diagonally weighted least squared models, χ^2^ test statistics tend to over-reject models (i.e., significant χ^2^ *p*‐values;^74^.

## Results

### Descriptive statistics

The descriptive statistics for, and correlations between, each variable are shown in Table 1. The descriptive statistics show that participants were experiencing high levels of psychological distress. Psychological distress was positively associated with COVID-19 concerns and avoidant coping; and was negatively associated with social support, psychological flexibility, and approach coping strategies.

**Table 1:** Descriptive statistics for each variable in the path analysis and bivariate Pearson correlations between each variable.

Variable	M	SD	95% CI		Correlations				
			Lower	Upper	1	2	3	4	5
1. COVID-19 concerns	87.38	11.82	86.15	88.62					
2. Social support	59.77	16.09	58.09	61.44	.18**				
3. Psychological flexibility	75.94	22.78	73.57	78.32	− .07	.40**			
4. Approach coping	29.83	7.14	29.11	30.60	.23**	.36**	.28**		
5. Avoidant coping	23.65	6.40	22.98	24.31	.10*	− .27**	− .63**	.03	
6. Psychological distress	26.20	9.91	25.17	27.23	.13*	− .38**	− .70**	− .12*	.72**

*p < 0.05; **p < 0.01.

### Mediation model

**Figure 2:**
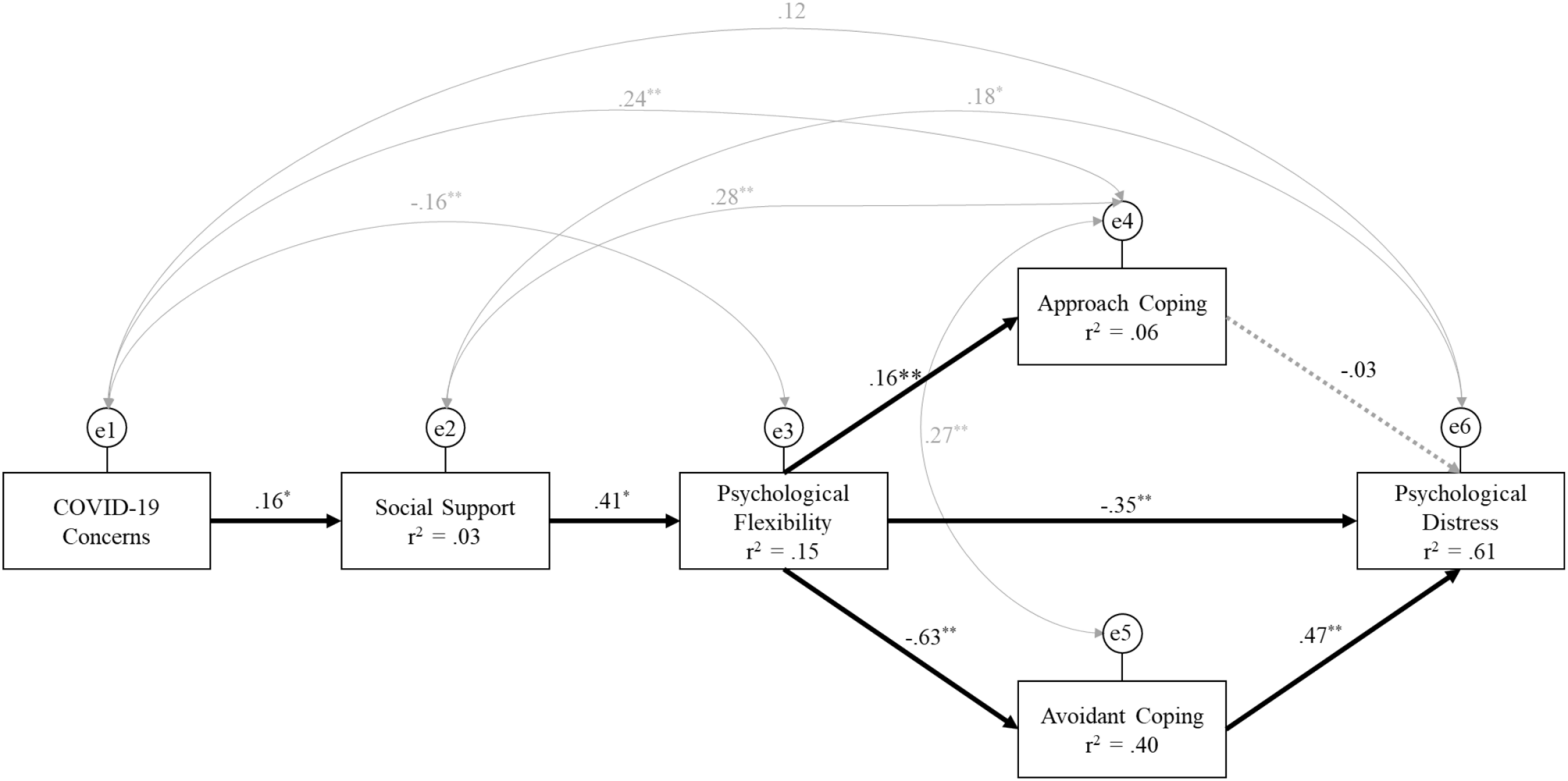
The final model predicts the pathway from COVID-19 concerns, social support, psychological flexibility, approach coping styles, avoidant coping styles, and psychological distress. Solid lines represent significant pathways, and dotted lines represent non-significant pathways. All values are standardised beta weights. *p < 0.05; **p < 0.01; ***p < 0.001.

A serial-parallel mediation analysis was conducted to identify if the relationship between concerns about COVID-19 and psychological distress (i.e., K10) is mediated by social support, psychological flexibility, and coping styles. The final model (Fig. 2) showed excellent model fit, χ^2^ (2) = 1.91, *p* = 0.390; RMSEA < 0.001, *p* = 0.645; AGIF = 0.98; TLI = 0.997. The model estimated that 60.7% of the variance in psychological distress (i.e., K10) was accounted for by the predictor variables (i.e., psychological flexibility, approach coping and avoidant coping). Psychological flexibility accounted for 39.6% of the variance in avoidant coping and 6.4% of the variance in approach coping. Social support explained 15.2% of the variance in psychological flexibility and COVID-19 concerns explained 2.6% of the variance in social support. The hypothesised model was largely supported by the data with the model accounting for a large percentage of the variance in the psychological distress of participants.

We also tested two additional models. The second model tested if psychological flexibility directly leads to social support (i.e., social support and psychological flexibility were switched in the model). However, an adequate model fit could not be attained, χ^2^ (2) = 1.91, *p* < *0.001*; RMSEA = 0.141 *p* = 0.007; AGIF = 0.84; TLI = 0.85. We also tested a moderated mediation model by adding a social support × psychological flexibility interaction. However, adequate model fit could not be attained, χ^2^ (4) = 92.29, *p* < *0.001*; RMSEA = .0.25 *p* < 0.001; AGIF = 0.55; TLI = 0.77. Therefore, the hypothesised model was retained. The confirmed hypothesised model is presented in Fig. 2.

### Direct effects

As presented in Fig. 2, all the direct paths, except for the path from approach coping styles to psychological distress were significant. The direct path from COVID-19 concerns to social support was positive and significant, B = 6.26, BC 95% CI [2.22, 10.38], *p* = 0.002. The direct path from the social support to psychological flexibility was positive and significant, B = 0.59, BC 95% CI [0.46, 0.74], *p* = 0.001. The direct path from psychological flexibility to approach coping styles was positive and significant, B = 0.05, BC 95% CI [0.02, 0.08], *p* = 0.002; and to avoidant coping was negative and significant, B = − 0.18, BC 95% CI [− 0.20, − 0.15], *p* = 0.001. The direct path from psychological flexibility to psychological distress was negative and significant, B = .-0.15, BC 95% CI [-0.19, -0.11], *p* = 0.001; and the direct path from avoidant coping styles to psychological distress was positive and significant, B = 0.72, BC 95% CI [0.59, 0.85], *p* = 0.001. The direct path from approach coping to psychological distress was non-significant, B = -0.04, BC 95% CI [< 0.001, < 0.001], *p* = 0.380.

### Indirect effects

The results of each indirect path are presented in Table 2, all indirect paths were significant (i.e., *p* ≤ 0.001 for all indirect pathways). The association between COVID-19 concerns and psychological flexibility was significantly mediated by social support. That is when individuals have a high number of COVID-19 concerns they have significantly higher social support, and their increased social support is in turn associated with increased psychological flexibility. The effect of social support on coping styles was mediated by psychological flexibility. This suggests that social support increases psychological flexibility and indirectly contributes to reducing avoidant coping strategies, and increasing approach coping strategies. Social support also indirectly reduced psychological distress through psychological flexibility and coping strategies. Psychological flexibility indirectly produced a significant reduction in psychological distress through avoidant coping strategies and approach coping strategies. This suggests that in addition to psychological flexibility’s direct effect of reducing psychological distress, psychological flexibility indirectly reduces psychological distress by decreasing the use of avoidant coping strategies and increasing approach coping strategies.

**Table 2:** The indirect pathways in the tested model (i.e., Fig. 2). Unstandardised Beta weights and standardised beta weights and their associated bias-corrected (BC) 95% confidence intervals.

Indirect pathways	B	BC 95% CI		β	BC 95% CI	
		Lower	Upper		Lower	Upper
CC → SS → PF***	3.71	1.35	6.63	0.07	0.02	0.12
CC → SS → PF → Avoid***	− 0.65	− 1.20	− 0.24	− 0.04	− 0.08	− 0.01
CC → SS → PF → Approach***	0.18	0.05	0.42	0.01	0.00	0.02
CC → SS → PF → $\frac{{{\text{Avoidant}}}}{{{\text{Approach}}}}$ → K10***	− 1.04	− 1.90	− 0.36	− 0.04	− 0.08	− 0.02
SS → PF → Approach***	− 1.04	− 0.14	− 0.08	− 0.26	− 0.33	− 0.20
SS → PF → Avoid***	0.03	0.01	0.05	0.07	0.02	0.12
SS → PF → $\frac{{{\text{Avoidant}}}}{{{\text{Approach}}}}$ → K10***	− 0.17	− 0.21	− 0.13	− 0.27	− 0.33	− 0.21
PF → $\frac{{{\text{Avoidant}}}}{{{\text{Approach}}}}$ → K10***	− 0.13	− 0.16	− 0.10	− 0.30	− 0.36	− 0.23

*p < 0.05; **p < 0.01; ***p < .001.

CC = COVID-19 Concerns; SS = Social Support; PF = Psychological flexibility, Avoid = Avoidant coping; Approach = Approach coping; and K10 = Psychological distress.

## Discussion

Our research aimed to test a novel model to show the pathway between experiencing stressful events (i.e., COVID-19) and psychological distress through social support, psychological flexibility, and coping mechanisms. Our research uniquely identified that having more COVID-19 concerns resulted in increased social support. This suggests that during stressful events, individuals are actively seeking social support, despite having strict social restrictions in place. We also confirmed that a direct positive association exists between social support and psychological flexibility and replicated recent findings from a Jordanian sample^60^.

One novel finding in our study was the mediating role of coping mechanisms between psychological flexibility and psychological distress. Our results partially confirm that psychological flexibility might influence the type of coping an individual will employ during stressful events. Specifically, individuals with higher levels of psychological flexibility are more likely to increase their approach coping strategies. However, the most notable finding is that high levels of psychological flexibility have a strong effect on reducing avoidant coping strategies. This suggests that psychological flexibility might not be a coping mechanism, in and of itself, but could determine the type of coping strategies that will or will not be employed. Our results show that during COVID-19, seeking social support from others can improve psychological flexibility, allow individuals to utilise adaptive coping strategies and reduce avoidant coping strategies, and indirectly reduce their levels of psychological distress.

### COVID-19 related concerns and perceived social support

Our results show that high concerns about COVID-19 are associated with higher perceived social support. This result shows that having concerns about the current stressful circumstances leads to a higher perception of social support levels, presumably by actively seeking more social support to aid individuals in dealing with their stress-inducing concerns.

Recent research shows that social restrictions do not necessarily lead to decreased social support^19^. Individuals who perceived a lack of social support before the pandemic do indeed perceive a lack of social support during COVID-19 related restrictions. However, individuals who had well-established sources of social support before the pandemic, continue to perceive high levels of social support during the COVID-19 related restrictions. Our results support the notion that individuals are more likely to seek social support during the COVID-19 pandemic by utilising social media and online services^20–22^. These ways of receiving social support seem to be as effective in producing enhanced perceptions of social support as face-to-face contact.

These results are important to consider because perceived social support is an important protective factor against psychological distress in response to stressful events^11^. Moreover, it is not necessarily an objective assessment of a person’s social support network, but the perceived quality of the social support they receive that is protective against psychological distress^17^. Our results highlight that even in times of considerable social restrictions, attaining satisfactory social support is achievable, and individuals may have sought more social support than usual as a strategy to cope with the stressful situation.

### Perceived social support and psychological flexibility

Consistent with existing literature^60^, our results suggest that increased social support is associated with increased psychological flexibility. Specifically, we showed that perceived social support is an important factor in facilitating psychological flexibility and the subsequent decrease in psychological distress. By examining the relationship between social support and psychological flexibility, we have also provided evidence that psychological flexibility by itself is not always sufficient to reduce psychological distress^24^. Thus, it is important to identify additional factors that work in unison with psychological flexibility to successfully alleviate distress. One possible explanation is that individuals who perceive a lack of social support are preoccupied with their feelings of loneliness, which consequently hinders their ability to adapt to changing circumstances, rendering these individuals psychologically inflexible. Examining the relationship between social support and psychological flexibility is important to identify additional factors that work in unison with psychological flexibility to successfully alleviate distress.

### Psychological flexibility and coping strategies

As part of the novel mediation model presented in this paper, the mediating role of coping styles in the relationship between psychological flexibility and distress was supported. Specifically, the role of avoidant coping style was especially pronounced, with lower psychological flexibility associated with higher use of avoidant coping, and avoidant coping, in turn, contributed to higher psychological distress. Higher psychological flexibility was associated with less avoidant and more approach coping, with no significant relationship between approach coping and distress. These results corroborate recent findings by Dawson and Golijani-Moghaddam^33^ who also found avoidant coping as a significant factor in determining the relationship between psychological inflexibility and distress. Specifically, they found that psychological inflexibility indirectly increased psychological distress and decreased psychological wellbeing when an individual employed an avoidant coping style. Their findings suggest that lower psychological flexibility can result in a higher prevalence of avoidant coping styles which in turn elevate psychological distress. Our results provide support for their theory that psychological flexibility, in itself, might not be a coping mechanism but rather could increase the likelihood of a specific coping mechanism employed by an individual during stressful events^33^. Our results show that during stressful events, when individuals have high levels of psychological flexibility, they are significantly less likely to employ avoidant coping styles resulting in a reduction in psychological distress.

Previous literature suggested that psychological flexibility is protective against negative emotions, can improve adjustment to stressful situations, and improve mental health^29–31^. Others also found an indirect association between psychological *inflexibility* and psychological well-being through avoidant and approach coping strategies (e.g.,^33,58^). Building on this knowledge, our results indicate that psychological flexibility influences the type of coping strategies individuals will employ under stressful situations (e.g., during COVID-19). For example, when an individual is psychologically flexible this will lead them to reduce avoidant coping strategies and adopt more approach coping strategies. However, the reduction of avoidant coping strategies is more important for reducing psychological distress than employing more approach coping strategies. Our findings contribute to extending theoretical knowledge by specifically examining the role of psychological flexibility, as opposed to inflexibility, which has been the focus of previous research. For example, where previous literature has demonstrated the role of psychological *inflexibility*, we have extended these findings by showing that psychological *flexibility* enhances approach coping, reduces avoidant coping strategies, and indirectly decreases psychological distress. These findings hold important theoretical implications for how we conceptualise and understand psychological flexibility as a facilitator of adaptive and maladaptive coping strategies and its indirect impact on psychological distress during stressful life events and warrants the continued investigation of psychological flexibility.

### Limitations

It is important to consider that the participants in our sample were predominantly female (i.e., 74.4%). This raises a concern regarding the generalisability of results to the broader general population. To avoid a gender-related bias, we examined gender differences in all variables in our study and found no significant differences. Another limitation is the study’s cross-sectional design, which enabled us to collect data from only a specific moment of participants’ experiences rather than following processes over time. It would be profitable to conduct longitudinal research to provide further support for our suggested model and consequently target the enhancement of social support and psychological flexibility to reduce psychological distress.

## Conclusion

We have provided a novel model to explain the pathway between COVID-19 concerns and psychological distress. Our model has provided two unique contributions. First, we identified a direct association between social support and psychological flexibility. Whereby, higher levels of social support facilitate higher levels of psychological flexibility. Second, we have provided support to the argument that psychological flexibility might not be a coping mechanism but may facilitate the type of coping strategies individuals employ^33^. Specifically, we showed that high levels of psychological flexibility reduce the usage of avoidant coping strategies and increase approach coping strategies. Within the context of COVID-19, we have shown that social support, psychological flexibility and the types of coping mechanisms individuals employ have an impact on their levels of psychological distress. However, we argue that the proposed model could be applied to other stressful events. There are important clinical and therapeutic implications for acknowledging the impact of social support, psychological flexibility, and specific coping style mechanisms. For example, psychosocial interventions could be developed to focus on building psychological flexibility to indirectly reduce avoidant coping strategies, especially in instances where it is difficult to directly change a person’s avoidant or maladaptive coping strategies. Based on our findings, by improving psychological flexibility, individuals will be more inclined to reduce avoidant coping and adopt more approach coping strategies. Lastly, to strengthen the model’s empirical validity, future research should aim to replicate our model by substituting COVID-19 concerns with other stress-inducing events or circumstances.
